# Ellagic Acid Inclusion Complex-Loaded Hydrogels as an Efficient Controlled Release System: Design, Fabrication and In Vitro Evaluation

**DOI:** 10.3390/jfb14050278

**Published:** 2023-05-16

**Authors:** Chengqun Yu, Abid Naeem, Yali Liu, Yongmei Guan

**Affiliations:** 1 Key Laboratory of Modern Preparation of Traditional Chinese Medicines, Ministry of Education, Jiangxi University of Chinese Medicine, Nanchang 330004, China; yuchengqun@jxutcm.edu.cn (C.Y.); 20214003@jxutcm.edu.cn (A.N.); 2 Key Laboratory of Pharmacodynamics and Safety Evaluation, Health Commission of Jiangxi Province, 1688 Meiling Road, Nanchang 330006, China; 3 Key Laboratory of Pharmacodynamics and Quality Evaluation on Anti-Inflammatory Chinese Herbs, Jiangxi Administration of Traditional Chinese Medicine, Nanchang Medical College, 1688 Meiling Road, Nanchang 330006, China

**Keywords:** hydrogels, polyphenol, phytoconstituents, inclusion complexation, anti-oxidants, bioavailability

## Abstract

Oxidants play a crucial role in the development of oxidative stress, which is linked to disease progression. Ellagic acid is an effective antioxidant with applications in the treatment and prevention of several diseases, since it neutralizes free radicals and reduces oxidative stress. However, it has limited application due to its poor solubility and oral bioavailability. Since ellagic acid is hydrophobic, it is difficult to load it directly into hydrogels for controlled release applications. Therefore, the purpose of this study was to first prepare inclusion complexes of ellagic acid (EA) with hydroxypropyl-β-cyclodextrin and then load them into carbopol-934-grafted-2-acrylamido-2-methyl-1-propane sulfonic acid (CP-*g*-AMPS) hydrogels for orally controlled drug delivery. Fourier transform infrared spectroscopy (FTIR), X-ray diffraction (XRD), scanning electron microscopy (SEM), thermogravimetric analysis (TGA), and differential scanning calorimetry (DSC) were used to validate ellagic acid inclusion complexes and hydrogels. There was slightly higher swelling and drug release at pH 1.2 (42.20% and 92.13%) than at pH 7.4 (31.61% and 77.28%), respectively. Hydrogels had high porosity (88.90%) and biodegradation (9.2% per week in phosphate-buffered saline). Hydrogels were tested for their antioxidant properties in vitro against 2,2-diphenyl-1-picrylhydrazyl (DPPH) and 2,2′-azino-bis (3-ethylbenzothiazoline-6-sulfonic acid) (ABTS). Additionally, the antibacterial activity of hydrogels was demonstrated against Gram-positive bacterial strains (*Staphylococcus aureus* and *Escherichia coli*) and Gram-negative bacterial strains (*Pseudomonas aeruginosa*).

## 1. Introduction

Ellagic acid (EA), a polyphenol with the molecular formula C_14_H_6_O_8_, is found in a wide variety of fruits and nuts, such as pomegranates, strawberries, blueberries, and walnuts. It possesses strong anti-oxidant, anti-inflammatory, anti-viral, anti-microbial, anti-teratogenic, hepatoprotective, and anti-tumor properties, particularly in regard to colon cancer, esophageal cancer, breast cancer, and hepatocellular carcinoma [1]. Its low oral bioavailability and consequently low therapeutic potential is due to a combination of factors, including its low aqueous solubility, its permeability in the gastrointestinal tract (GIT), its permanent binding to DNA and proteins, and its first-pass metabolism. Furthermore, EA is rapidly absorbed in the GIT and eliminated within 2 to 6 h, depending on the composition of the food [2].

Previous studies investigated the in vivo and in vitro bioavailability of EA in humans, which revealed substantial interindividual variation (due to the low solubility of free EA in gastric conditions, the nature of the precursor, and insufficient intestinal absorption). Moreover, despite high intakes of free EA, plasma levels of EA were consistently low [3]. Research has demonstrated that EA undergoes extensive metabolism in the large intestine by the gut microbiota, resulting in urolithins that are 25–80 times more bioavailable and much more readily absorbed than EA [4,5]. Presently, research on ellagic acid focuses primarily on the distribution, extraction and purification, analysis of its content, and pharmacological properties of the compound. However, relatively few studies have been conducted on its formulations, including ellagic acid microspheres, phospholipid complexes, and nanoparticles [6,7,8,9]. These data have prompted an increase in interest in developing delivery systems, which are capable of increasing the solubility and bioavailability of EA in water, maintaining its structural integrity, and ensuring its controlled release at a physiological target [10].

Cyclodextrins (CDs) are cyclic oligosaccharides, consisting of (α-1,4) linked α-d-glucopyranose monomers. They have a lipophilic cavity, a ‘molecular cage,’ which may combine with the lipophilic part of the molecule via non-covalent interactions, while their hydrophilic surface aids in water solubilization [11]. In addition, this molecular inclusion enhances shelf life by protecting it against oxidation, light-induced decomposition, and heat-induced transformation. Unlike most other CDs, α-cyclodextrin lacks a sufficiently large cavity in which to encapsulate the majority of molecules, while γ-cyclodextrin does not offer economic viability because of its high price [12]. Therefore, β-cyclodextrin (β-CD) represents the most common form of the derivative. 2-Hydroxypropyl-β-cyclodextrin (HP-βCD) is an improved form of naturally occurring β-cyclodextrin, exhibiting enhanced safety characteristics. HP-βCD, which is generally recognized as safe by the FDA, is a water-soluble oligosaccharide that has shown promising results in increasing the solubility of various molecules [13].

Drug release can be controlled through the use of polymers that have known release profiles. As a result, drug release rate fluctuations may be minimized, and effective delivery can be achieved [14,15]. A controlled drug release system typically delivers the drug, over time, to the target area at a predetermined rate so as to maintain the concentration of the drug and to optimize therapeutic effectiveness [16]. Hydrogels are characterized by a cross-linked network of hydrophilic polymers that can absorb water without becoming soluble in it [17]. Hydrogels have found extensive applications in agriculture, biotechnology, food, and pharmacy. As a result of their swelling properties, hydrogels can absorb and release substantial amounts of drugs. There is a direct correlation between the swelling behavior of the hydrogel and its release rate [18]. Carbopol polymers are generally considered to be safe by the US Food and Drug Administration (FDA) [19]. In addition to being a hydrophilic polymer, carbopol-934 becomes highly ionized upon neutralization, causing electrostatic repulsion among polymer chains to produce a gel. The high stability, compatibility, and low toxicity of carbopol make it ideal for use in oral preparations, such as suspensions, tablets, and topically applied formulations [20]. 2-acrylamido-2 methylpropane sulfonic acid (AMPS) possesses high hydrophilicity and is commonly utilized in hydrogel formulations. AMPS shows pH-independent swelling characteristics due to the presence of sulfonic groups, which are easily ionized and dissociated at different pH values [21]. Furthermore, the increase in AMPS monomer concentration increases the swelling ratio of the hydrogels, owing to the presence of more ionizable sulfonic groups [22]. Ethylene glycol dimethacrylate (EGDMA) is a chemical crosslinker widely utilized for crosslinking of different polymers and monomers [23].

The aim of this study was to successfully encapsulate a hydrophobic molecule (ellagic acid) in hydrogels for orally controlled drug delivery to improve its bioavailability. Because most of the hydrophobic drugs cannot be very easily loaded into hydrogels, the first inclusion complexes were formed and then loaded in the hydrogels. This type of formulation provides a basis for encapsulating other hydrophobic phytochemicals/drugs in hydrogels for controlled release purposes for improving their bioavailability. In this study, first, the inclusion complexes of the hydrophobic drug (ellagic acid) with 2-hydroxypropyl-β-cyclodextrin (EIC), were developed to improve the solubility and stability of the drug. Then, these inclusion complexes were loaded inside carbopol-934-grafted-AMPS hydrogels. Various characterization techniques were utilized for characterizing the inclusion complexes and hydrogels, such as Fourier transform infrared spectroscopy (FTIR), differential scanning calorimetry (DSC), X-ray diffraction (XRD), thermogravimetric analysis (TGA), hydrogel porosity and sol–gel fraction, and biodegradation. The swelling characteristics and drug release behavior of the formulated hydrogels were determined using phosphate buffer (pH 7.4 and 1.2). Furthermore, the antioxidant (ABTS and DPPH) and antimicrobial activities (*Pseudomonas aeruginosa*, *Staphylococcus aureus*, and *Escherichia coli*) of ellagic acid inclusion complexes-loaded hydrogels were also investigated. As a result of their excellent water absorption, biodegradability, and controlled release behavior, these hydrogels are capable of being used as controlled oral drug delivery systems for hydrophilic and hydrophobic drugs, respectively.

## 2. Materials and Methods

### 2.1. Materials

Ellagic acid (EA; MW: 302.19 g/moL; Lot#R016669), ethylene glycol dimethacrylate (EGDMA; MW: 198.22 g/moL; Lot#335681), ammonium persulfate (APS; Lot#MKCM6448), and 2-acrylamido-2-methyl-1-propanesulfonic acid (AMPS; MW: 207.25 g/moL; Lot#STBK4291) were obtained from Sigma-Aldrich, Saint Louis, MI, USA. Sodium bisulfite (SHS; Lot#G2223206) was from Shanghai Aladdin Biochemical Technology, Shanghai, China. HP-βCD (HPβCD; MW:1541.5 g/moL; Lot#O1027C), cefepime HCL (Lot#N1118A), ABTS (Lot#A0807A), and DPPH (Lot#UFQ2I-WL) were acquired from Meilune Biological Company (Dalian, China). Carbopol-934 (CP; MW: 72.06 g/moL; Lot#C12467657) was obtained from Macklin Biochemical Co., Ltd. (Shanghai, China).

*Escherichia coli* (*E. coli*: ATCC25922HBJZ087), *Staphylococcus aureus* (*S. aureus*: ATCC25923HBJZ005), and *Pseudomonas aeruginosa* (*P. aeruginosa*: ATCC27853HBJZ017) were acquired from Qingdao Hope Biotechnology, Co., Ltd., Qingdao, China.

### 2.2. Synthesis of HP-βCD and Ellagic Acid Inclusion Complexes (EIC)

The EIC was prepared by using the mass ratio of HP-βCD to ellagic acid of 2:1 [24]. Briefly, HP-βCD was weighed and dissolved in a specified amount of ultrapure water, and ellagic acid was dissolved in an appropriate amount of anhydrous ethanol. The ellagic acid solution was then gradually added to the HP-βCD solution, and it was mixed well at 350 rpm/min for 72 h. A thorough mixing procedure was followed by centrifugation and storage in a –20 °C refrigerator for approximately 12 h. In the final step, the obtained sample was freeze-dried for two days in order to obtain pure HP-βCD and ellagic acid inclusion complexes.

### 2.3. Fabrication of CP-g-AMPS Hydrogels

**Figure 1:**
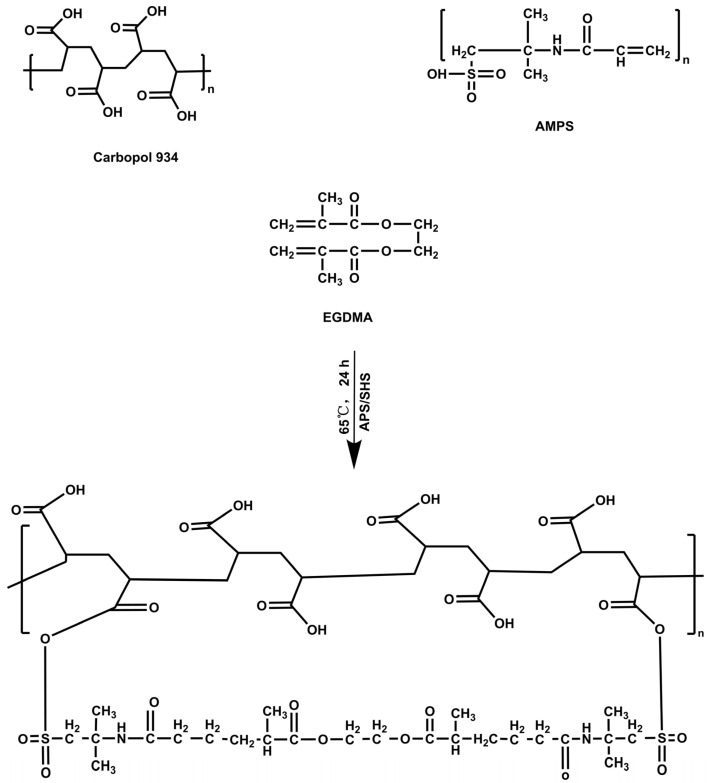
Proposed chemical structure of CP-*g*-AMPS hydrogels.

The free radical polymerization technique was used for the synthesis of hydrogels, possessing slight modifications [22]. Briefly, carbopol-934, AMPS, EGDMA, and APS/SHS (APS was used as an initiator, and SHS was used as a co-initiator of the reaction) were weighed and placed separately in their corresponding bottles, as shown in Table 1. The labeled vials were filled with sufficient water and stirred vigorously to obtain a clear solution. The carbopol-934 was continuously stirred at 40 °C until it was well blended. A homogeneous mixture of APS/SHS and AMPS was prepared by mixing it evenly, and then it was added to the carbopol-934 solution. After mixing, the mixture was ultrasonically processed, and nitrogen was applied for 30 min to remove air bubbles. Following this, EGDMA was poured dropwise into the mixture. The final mixture was transferred to a water bath (50 °C), and it was slowly heated until the temperature reached 65 °C. After 24 h, the hydrogels were cut into discs of 8 mm, and they were vacuum dried at 40 °C for a week. Figure 1 illustrates the proposed chemical structure for CP-*g*-AMPS.

#### EIC Loading into Hydrogels

The EIC was loaded into the synthesized hydrogels by a swelling–diffusion procedure [25]. Briefly, 1% EIC solution was prepared in buffer pH 7.4, and, then, unloaded hydrogel discs (dried) were placed in it and stirred slowly for three days. Thereafter, the hydrogel discs were taken out, allowed to dry fully, weighed, and the amount of EIC-loaded in the hydrogel was determined by subtracting unloaded hydrogel weight from EIC-loaded hydrogel weight. In addition, UV-spectrometry was used to confirm the drug loading by extracting the drug from the hydrogel and analyzing it. Briefly, the drug-loaded hydrogel discs were immersed in freshly made 25 mL of pH 7.4 phosphate buffer for a period of 5 h. We changed the phosphate buffer solution after a predetermined time to a new 25 mL solution. Repeated testing was conducted until no drug was detected in the solution. A calibration curve for ellagic acid was used to measure the concentration of ellagic acid in the solution. A UV-visible spectrophotometer was used to determine the absorbance at a wavelength of 254 nm.

(1) EIC loading=EIC loaded hydrogels−Unloaded hydrogels

### 2.4. In Vitro Characterization

#### 2.4.1. FTIR Spectroscopy

Drug–formulation interactions were examined with the attenuated total reflectance method using a FTIR spectrometer (Spectrum Two FTIR Spectrometer, Perkin Elmer, Buckinghamshire, UK). The spectra of loaded and unloaded formulations and their components were acquired over a scanning range of 400 cm^−1^ to 4000 cm^−1^.

#### 2.4.2. Thermal Analysis

Thermal stability of the samples was assessed through the use of a thermogravimetric analyzer (TG/DTA6300, Seiko, Tokyo, Japan) and a differential scanning calorimeter (DSC 4000, Perkin Elmer, UK). An analysis of weight change with respect to temperature was conducted using a thermogravimetric instrument. A reference standard was used to calibrate the weight profile at the beginning. Carbopol-934, AMPS, EIC, and their respective formulations (0.4 to 6 mg) were individually tested in aluminum pans. A scanner operating at a temperature of 10 °C/min and a flow rate of 10 mL/min of inert nitrogen was used to measure the percentage weight loss. AMPS, EIC, carbopol-934, and synthesized formulations were analyzed using DSC to determine their melting points. A sapphire standard was employed for calibrating calorimeters for heat capacity. A standardization of cell constants and temperatures was achieved through the use of indium.

#### 2.4.3. X-ray Diffraction

Crystallinity of the samples was determined by a diffractometer (TD-3500 X-ray diffractometer, Dandong, China), with CuKα irradiations at 30 kV and 20 mA. Scan speeds were set at 2 degrees per minute and 2θ between 10° and 60°. The crystallinity of an object can be determined by its peak. Normally, the presence of sharp and intensive peaks indicates the crystallinity of the sample. In contrast, diffuse peaks indicate an amorphous nature, as demonstrated by synthesized hydrogels. Measurements were conducted on carbopol-934, EIC, HP-βCD, ellagic acid, and unloaded and EIC-loaded hydrogels.

#### 2.4.4. Scanning Electron Microscopy

The morphology, microstructural properties, and porosity of the hydrogel were investigated with the help of scanning electron microscopy (Quanta 250, Eindhoven, The Netherlands). The dried samples were first coated on aluminium stubs, and then they were sputtered with gold, and then they were evaluated in a cross-section, using an accelerated current of 15 kV.

#### 2.4.5. Mechanical Characteristics

Tensile strength (TS) and elongation-at-break (EAB) of the hydrogels were measured for each formulation at 1 mm/s using a TA.XT plus texture analyzer (SMS TA.XT plus, Stable Micro Systems, Godalming, UK), which was equipped with a stainless steel cylindrical probe (P/5S) [26]. TS and EAB can be calculated using the following equations.

(2) $$\mathrm{TS}=\frac{\mathrm{Fm}}{\mathrm{Th}}$$

(3) $$\mathrm{EAB}=\frac{\sqrt{D^{2}+R^{2}}}{R}-1$$

Fm represents the probe force, and Th represents the hydrogel thickness. D is the displacement of the probe after it first contacts the hydrogel, and R is the radius of the orifice plate.

#### 2.4.6. Sol-Gel Fraction Determination

Sol-gel fraction analysis was conducted on the synthesized hydrogels with the aim of determining the levels of uncrosslinked and crosslinked constituents [27]. The hydrogels are composed of two different parts/portions: a gel fraction, which is an insoluble component, and a sol fraction, which is a soluble component. The Soxhlet extraction method was used to conduct the sol-gel fraction analysis. Unloaded hydrogels were added to a flask containing deionized water in a certain amount. The flask was equipped with a condenser. At 85 °C, the extraction was carried out for 14 h. Afterwards, the disc was extracted, dehydrated in a vacuum oven, and then weighed again. The given equations were used for determining the sol–gel fraction of hydrogel [28].

(4) $$\mathrm{Sol}\;\mathrm{fraction}\;\%=\frac{S1-S2}{S2\;}\times 100$$

(5) Gel fraction=100−sol fraction

The weight of a dried hydrogel (after extraction) is indicated by S1, whereas that of a fresh hydrogel is shown by S2.

#### 2.4.7. Porosity Study

The porosity of each hydrogel formulation was assessed using a solvent replacement approach. Once the dried hydrogels (K1) had been accurately weighed, they were placed in ethanol (pure) for five days. After five days, the discs were lifted out of the ethanol, gently dried, and then weighed again (K2). Additionally, the diameter of the hydrogel disc, as well as its thickness, were measured. The equation given below was used to determine porosity [29].

(6) $$\mathrm{Porosity}\;\mathrm{percentage}\;\left(\%\right)=\frac{K2-\;K1\;}{\rho v\;}\times 100$$

Here, ρ represents the absolute density of ethanol, and V indicates swollen hydrogel volume.

#### 2.4.8. Polymer Network Parameters of CP-g-AMPS Hydrogels

The properties and structure of swollen hydrogels are determined by a number of important factors [30].

The diffusion coefficient (D) was calculated as follows:

(7) $$D=\pi {\left(\frac{h\cdot \theta }{4\cdot \mathrm{qeq}}\right)}^{2}$$

Whereas, qeq indicates equilibrium swelling, θ indicates slope of swelling curves, and h indicates disc thickness.

The polymeric volume fraction (V2, s) was determined as follows.

(8) $$V2,\;s=\frac{1}{\mathrm{Veq}\;}$$

Molecular weight across crosslinking connections (Mc) was determined as follows.

(9) $$\;\mathrm{Mc}\;=\frac{\mathrm{dp}\;\mathrm{Vs}\left({V\;}^{\frac{1}{3}}2,s-V2,\frac{s}{2}\right)}{\ln\left(1-V2,s\right)+V2,s+{\chi V}^{2}2,s}\;\;\;\;\;\;$$

Whereas dp corresponds to polymer density, χ is the Flory-Huggins solvent polymer interaction parameter, and Vs refers to the volume of the solvent.

The polymer solvent interaction parameter (χ) can be determined with the following equation.

(10) $$\chi =\frac{\ln\left(1-V2,s\right)+V2,s}{V^{2}2,s\;}$$

where; V2,s corresponds to the hydrogel volume after swelling.

The number of intercrosslink repeating units (N) were estimated by the following equation:

(11) $$N=\frac{2\mathrm{Mc}}{\;\mathrm{Mr}\;}$$

Mr denotes molar mass of the repeating units and is determined using the given equation:

(12) $$\mathrm{Mr}=\frac{\mathrm{mCPMCP}\;+\;\mathrm{mAMPSMAMPS}\;+\;\mathrm{mEGDMAMEGDMA}}{\mathrm{mCP}+\mathrm{mAMPS}+\mathrm{mEGDMA}}$$

where “m” represents masses, and “M” shows the molar masses of carbopol-934, AMPS, and EGDMA, respectively.

#### 2.4.9. Swelling Studies

Equilibrium-swelling ratios (ESR) were determined using phosphate buffers (pH 1.2 and 7.4) [31]. The weight of the hydrogels was measured after weighing and placing them in the buffers for a specified period of time. The weight of the hydrogel was measured and recorded at a number of different points until equilibrium was reached. The swelling percentage was calculated using the formula shown below.

(13) $$\mathrm{ESR}=\frac{\mathrm{Yt}\;-\;\mathrm{Yi}}{\;\mathrm{Yi}\;}\times 100$$

The weight at time t is indicated by Yt, and the initial weight of the hydrogel is given by Yi.

#### 2.4.10. In Vitro Release and Kinetics

Synthesized hydrogels were investigated for drug release in vitro at two pH levels, pH 1.2 and 7.4 [32]. Briefly, dried EIC-loaded hydrogel discs were immersed in pH 1.2 and 7.4 buffer solutions in a USP dissolution apparatus type II at 37 °C and 50 rpm. Each time, an aliquot of 5 mL was collected, and the same volume of fresh medium was infused into the sample at predetermined intervals. Samples were analyzed in triplicate using an ultraviolet-visible spectrophotometer (T6 New Century, Beijing, China) at a wavelength of 254 nanometers (the standard wavelength for ellagic acid).

(14) $$\mathrm{Percentage}\;\mathrm{drug}\;\mathrm{release}=\frac{\left(\mathrm{drug}\;\mathrm{released}\right)}{\left(\mathrm{drug}\;\mathrm{loaded}\;\right)}\times 100$$

Among the most important parameters that can affect the rate of drug release in hydrogels are polymer chain stretching, matrix swelling, drug type, and the pH of the medium in which the drug is released. Swelling of hydrogels is fundamentally explained by solvent diffusion, as it is proportional to the penetration of solvents into the hydrogels, which causes them to swell. The following release kinetic models were used to identify the pattern of drug release.

(15) Zero−order kinetics Ft=K0t

Ft represents the quantity of drug that has been released over time t, while K0 represents the apparent constant of the zero-order rate of drug release over time t.

(16) $$\mathrm{First}\;\mathrm{order}\;\mathrm{kinetics}\;\ln\left(1-F\right)=-K1t$$

The amount of drug that is released at time t is denoted by the symbol F, and the first-order drug release rate constant is denoted by k1.

(17) $$\mathrm{Higuchi}\;\mathrm{model}\;F=K2t^{\frac{1}{2}}$$

The Higuchi constant is denoted by the symbol K2 in this equation, while the release rate is denoted by the letter F.

(18) $$\;\mathrm{Korsmeyer}-\mathrm{Peppas}\;\mathrm{model}\;\frac{\mathrm{Mt}}{M¥}=K3t^{n}$$

The quantity of water that is absorbed into a system when it is in a state of equilibrium is expressed by the symbol M∞, whereas the quantity that is absorbed at a particular time is represented by the symbol Mt. The geometrical and structural features of gels are the basis for the K3 constant, and the exponent of drug release is denoted by n. 

#### 2.4.11. Biodegradation Study

Biodegradation study of the fabricated hydrogel was carried out at 37 °C using a pH 7.4 phosphate buffer [33]. At different intervals, precisely weighed hydrogel discs were immersed in a buffer solution for one, three, five, and seven days, respectively. After taking out the hydrogel discs at the designated time, the hydrogel discs were dried under vacuum at 40 °C, reweighed, and then put into the buffer solution. Degradation of hydrogels was calculated using the following equation [34].

(19) $$D=\frac{C1-C2}{C1}$$

D shows degradation, and C1 shows dry weight, while C2 shows after-immersion weight.

### 2.5. Antioxidant Studies

#### 2.5.1. DPPH Activity

The antioxidant activity of CP-*g*-AMPS hydrogel was measured using the DPPH free radical scavenging assay. After soaking a specified amount of sample in methanol, it was left at ambient temperature in the dark for 24 h. After that, all samples were added to the DPPH-methanol solution (0.1 M). Afterward, the mixture was thoroughly shaken and allowed to stand in the dark for 30 min. A UV-Vis spectrophotometer was used to measure the solution’s absorbance at 517 nm, and the DPPH scavenging activity was calculated accordingly [35].

(20) $$\mathrm{DPPH}\left(\%\right)=\frac{A0-\;A}{A0}\times 100$$

Absorbance values A0 and A indicate the control and sample, respectively.

#### 2.5.2. ABTS Activity

Hydrogels, containing EIC, were tested with the ABTS assay for their radical scavenging capability. The radicalization of ABTS was induced by adding 2.4 mm potassium persulfate (K_2_S_2_O_8_) to a solution of 7.4 mM ABTS. After the hydrogels and the ABTS solution had been thoroughly combined, the mixture was allowed to sit in an incubator at 37 °C for half an hour. An absorbance measurement was performed at a wavelength of 730 nm. The scavenging effect of ABTS is calculated using the following formula [36].

(21) $$\mathrm{ABTS}\;\mathrm{scavenging}\;\mathrm{effect}\;\left(\%\right)=\frac{A0-A1}{A0}\times 100$$

The absorbance of ABTS is A0, whereas the absorbance of the samples is A1.

### 2.6. Antibacterial Activity

Nutrient agar was formulated with purified water, and it was subsequently autoclaved (121 °C/15 psi) for 30 min. Agar medium was applied to small Petri plates and allowed to solidify for a few hours at room temperature before use. Bacterial strains of *Pseudomonas aeruginosa*, *Escherichia coli*, and *Staphylococcus aureus* were cultured for 24 h, and then they were swabbed and grown on Petri dishes. Each of these plates was divided into four groups: blank hydrogel, EIC-loaded hydrogel, cefipime (1 mg/mL, positive control), and negative control. Additionally, they were then incubated for 24 h at 37 °C before calculating the zone of inhibition (ZOI) [37]:

(22) $$\mathrm{Inhibition}\;\left(\%\right)=\frac{\mathrm{ZOI}\;\mathrm{of}\;\mathrm{test}\;\mathrm{sample}\;\left(\mathrm{mm}\right)}{\mathrm{ZOI}\;\mathrm{of}\;\mathrm{standard}\;\mathrm{drug}\;\left(\mathrm{mm}\right)}\times 100$$

### 2.7. Statistical Analysis

Mean and standard deviations were used to quantitatively describe the data. Two-way analysis of variance and Tukey’s post hoc test were used to find statistically significant differences between sets of neighbouring data. Statistical significance was determined by calculating the *p*-value, and the results were marked as follows: * *p* < 0.05, ** *p* < 0.01, and *** *p* < 0.001.

## 3. Results and Discussion

### 3.1. FTIR Analysis

**Figure 2:**
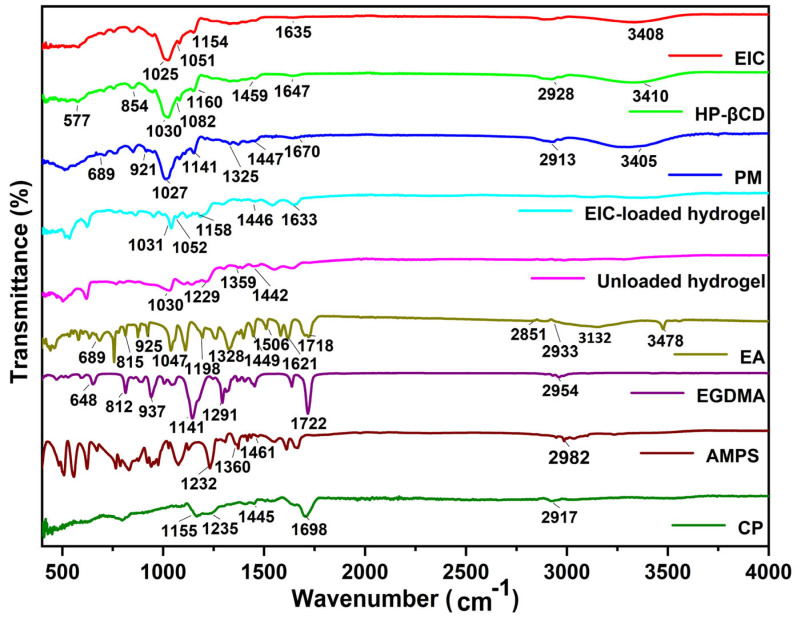
FTIR spectra of ellagic acid, EIC, HP-βCD, physical mixture (PM), CP, AMPS, EGDMA, unloaded, and EIC-loaded hydrogels.

The EA spectrum showed characteristic absorption bands similar to the ones previously described (Figure 2). A large sharp band at 3480 cm^−1^, and a broad absorption band at 3360–2840 cm^−1^, were assigned to the stretching vibrations of the -OH group. Absorption bands at 2933 cm^−1^ and 2851 cm^−1^ are due to asymmetric, as well as symmetric C–H, stretching vibrations for –CH_3_. Stretching of the carbonyl group appeared at 1718 cm^−1^. The bands observed at 1621 cm^−1^ and 1506 cm^−1^ are attributed to C=C–C vibrations, and bands at 1449 cm^−1^ are attributed to C–H bending vibrations. Whereas, 1328 cm^−1^ and 1198 cm^−1^ bands are due to C–O ester linkage stretching, and 1047 cm^−1^ bands are for aromatic C–H in-plane bending ester linkage. Bands at 925, 815, and 689 cm^−1^ are assigned to aromatic C–H out-plane bending [38]. In the case of CP, the FTIR spectrum showed bands in the range of 3000–1940 cm^−1^, indicating stretching vibrations due to an –OH group of carboxylic acid and intramolecular H-bonding. A characteristic band at 1698 cm^−1^ was assigned to carbonyl group (C=O) stretching vibrations. Bands in the range of 1460–1400 cm^−1^ were assigned to –C–O and –OH bending vibrations. Bands at 1250–1200 cm^−1^ were due to the bending of –CH in methylene, and a prominent peak at 1155 cm^−1^ indicates stretching due to C–C bonding [39]. The HP-βCD exhibited –OH stretching vibration with a band of 3410 cm^−1^, whereas C-H stretching vibration was symmetrical. The characteristic bands at 1160 cm^−1^ and 1030 cm^−1^ were attributed to the C–O stretching vibration arising from HP-βCD spectrum. AMPS exhibited stretching vibrations at 1461 cm^−1^, 1360 cm^−1^, 2981 cm^−1^, and 1230 cm^−1^, corresponding to CH_2_, –C–O, –CH, and S=O stretching vibrations, respectively [40]. EGDMA showed bands at 1153, 1291, and 1633 cm^−1^, corresponding to C–O and C=C stretching vibrations for both symmetrical and asymmetric ester chains, and it also showed a band at 1713 cm^–1^ for C=O stretching vibrations [41]. 

The EIC spectrum showed an -OH stretching vibration band at 3408 cm^−1^, while the –OH characteristic band of EA disappeared. The band at 1635 cm^−1^ corresponds to the C=O stretching vibration band of EA. The characteristic absorption band of benzene ring disappeared or decreased, and the band at 1025 cm^−1^ belongs to the C-O stretching vibration band of HP-βCD. The band at 1051 cm^−1^ corresponds to the aromatic C-H plane curved ester bond in EA. In summary, this shows that ellagic acid is incorporated into the cavity of cyclodextrin to form EIC. For the physical mixture PM, the band at 3405 cm^−1^ corresponds to the tensile vibration of -OH, the band at 1447 cm^−1^ belongs to the C-H bending vibration of EA, the band at 577, 854 cm^−1^ corresponds to the aromatic C-H bending vibration of EA, and the band at 1027 cm^−1^ corresponds to the C-O stretching vibration of HP-βCD. Unloaded hydrogel displayed a different spectrum when compared with its parent components. The bands at 1442 cm^−1^ indicate the presence of bending vibration of –C–O and -OH in carbopol-934, respectively. The bands at 1359 and 1229 cm^−1^ correspond to the stretching vibration of –C–O in AMPS and a symmetric stretching vibration of a S=O functional group, respectively. The new bands and functional groups indicate that the polymer is successfully crosslinked. FTIR analysis of EIC-loaded hydrogel was also performed to confirm the presence of the drug. The bands at 1633 and 1158 cm^−1^ represent the tensile vibration band of C=O in EIC and the C–H bond in aromatic group, respectively. The appearance of these bands indicates that EIC has been successfully loaded into the hydrogel.

### 3.2. Thermal Analysis

**Figure 3:**
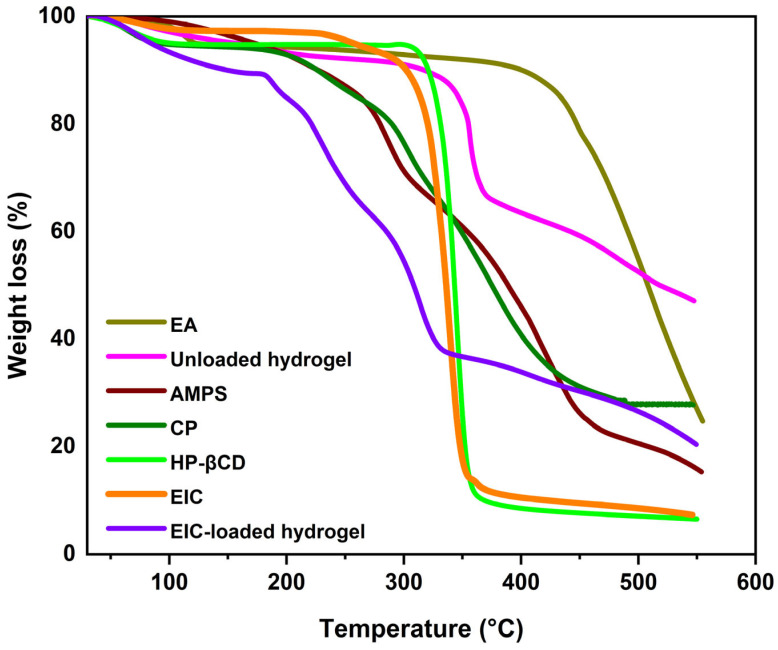
TGA of ellagic acid, HP-βCD, EIC, CP, AMPS, unloaded, and loaded hydrogels.

Hydrogels and polymers were thermally analyzed using TGA (Figure 3). The results showed that, as the CP temperature approaches 87 °C, it loses around 10% of its weight due to moisture evaporation. Similarly, the weight further drops by 21% when the temperature reaches 326 °C. The weight loss is a result of the decarboxylation of the polymer, resulting in unsaturated structures, as well as the depolymerization of the polymer, leading to degradation. CP began to degrade at 383 °C and eventually became completely decomposed [42]. TGA analysis of EA revealed a mass loss of 7.9% at 107 °C due to hydrogen bond separation from water. A subsequent weight loss of 36% was observed at 457 °C, in agreement with previous studies [43]. AMPS is shown to lose 6% of its weight at 208 °C based on thermogravimetric analysis. In the 210–250 °C temperature range, a further 20% weight loss was seen, indicative of the dehydration of AMPS. Additionally, the sulfonic acid group began to break down at 340 °C, with a weight loss of 20% recorded between 250 and 340 °C [44]. Consistent with prior findings, the current investigation indicated that HP-βCD samples lost weight below 80 °C due to water evaporation, whereas HP-βCD degradation occurred between 305 °C and 380 °C [45]. The inclusion complex exhibited a similar thermogram, and weight loss could be observed in three regions. The first and second regions, respectively, correspond to water loss and degradation of HP-βCD. There is also evidence for a slight modification in the degradation temperature of HP-βCD to inclusion complexes. These findings suggest that inclusion complexes have formed. It is evident that, when EA is complexed with HP-βCD, its thermal stability increases [46]. 

In the case of TGA analysis of EIC-loaded hydrogels, the initial weight loss was 15% due to dehydration at temperatures between 30 °C and 170 °C, followed by a 54% weight loss due to breakage of polymer bonds from 170 °C to 332 °C. Gradually, the polymer network began to degrade at 332 °C, continuing to degrade until all of the polymer skeletons had been completely destroyed. It was observed that the unloaded hydrogel lost 10% of its weight at 30–330 °C due to dehydration. In addition, it lost 26% at 330–362 °C due to a breakdown of the chemical bonds in the polymer. After 362 °C, the polymeric network gradually decomposes, and the polymer backbone degradation continues until complete degradation. Thermal profiles of hydrogels with a higher residual weight suggest that the polymeric matrix is more robust against thermal degradation over the entire temperature range investigated. Moreover, because of the increased strength and the contacts between the monomer and polymer, the degradation of the hydrogel occurs at higher temperatures and at a slower rate than with the individual reactants. Hydrogels’ thermal stability is evidenced by the formation of rigid networks, which represent superior stability.

**Figure 4:**
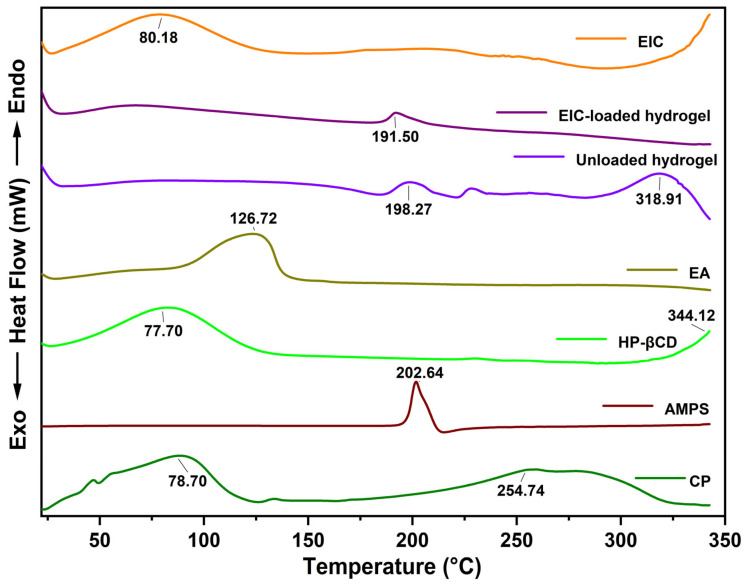
DSC thermogram of EIC, ellagic acid, AMPS, HP-βCD, CP, unloaded, and drug-loaded hydrogels.

The DSC thermogram of EA displayed an endothermic peak at approximately 126.72 °C, which could be attributed to the incorporation of water during crystallization [47]. In the DSC thermogram curve of HP-βCD, the melting point is approximately 344.12 °C, whereas the endothermic peak located at 77.70 °C shows water evaporation. Hydrogen bonds may be responsible for this transition between the -H group of water and the -OH group of HP-βCD [48]. The first endothermic peak in the DSC thermogram curve of AMPS can be seen at 193 °C, which represents the melting point of the substance. Decomposition is indicated by the endothermic peak at approximately 202.64 °C. CP exhibited two endothermic peaks at 78.70 °C and 254.74 °C, according to DSC thermogram measurements. Endothermic peaks may be attributed to water evaporation from CP, while the latter peak may be the result of anhydride formation [49]. A DSC thermogram analysis of the synthesized hydrogels revealed a higher glass transition temperature for the formulation as a result of higher intermolecular hydrogen bonding, which indicates a greater degree of compatibility between the individual components and a more rigid network structure. As a result, the developed polymeric hydrogel network shows a higher level of thermal stability (Figure 4).

### 3.3. XRD Study

**Figure 5:**
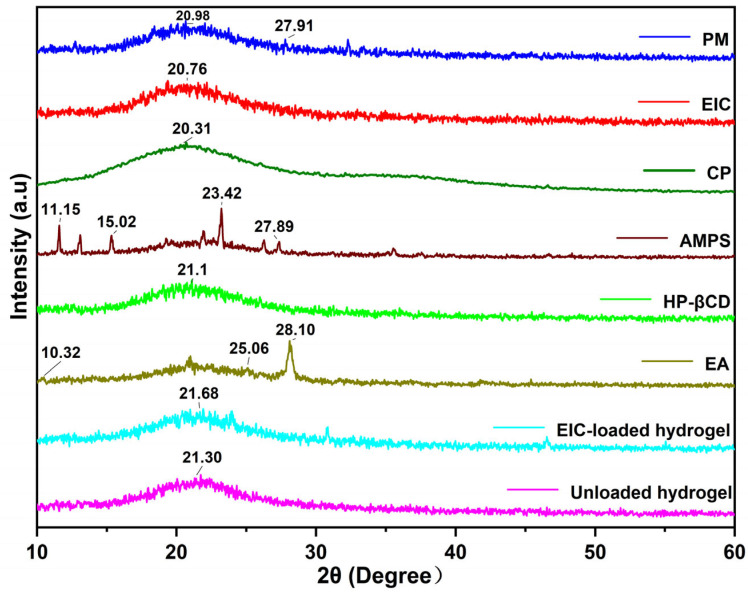
XRD of PM (physical mixture), EIC, CP, AMPS, HP-βCD, ellagic acid, EIC-loaded, and unloaded hydrogels.

Figure 5 illustrates XRD diffractograms of all components and hydrogel formulations. HP-βCD diffractogram showed a diffuse peak at 2θ of 21.1°, which suggested that HP-βCD is an amorphous form, which is similar to the results of others [50]. The XRD pattern of EA exhibited sharp peaks at diffraction angles (2θ) of 10.32°, 25.06°, and 28.10°, indicating the typical crystalline properties of EA [51]. Meanwhile, AMPS shows peaks at 2θ = 11.15°,15.02°, 23.42°, and 27.89°, which are suggestive of crystalline properties. It can be seen from the XRD pattern of physical mixture (PM) that the characteristic peak of ellagic acid can be found in the physical mixture, indicating that the characteristic peak of ellagic acid is simply superimposed, while the characteristic peak of ellagic acid does not appear in EIC, indicating that ellagic acid has been incorporated in the cavity of cyclodextrin to form the inclusion complex. The absence of new peaks indicated that no new crystals were formed between the EA and the carrier, maintaining the stability of the original components. The diffractogram of an unloaded hydrogel exhibits a broad diffuse peak at 2θ = 21.30°, whereas that of an EIC-loaded hydrogel exhibits only a broad diffuse peak at 2θ = 21.68°, with no other intense peaks of the drug seen in this region. This may have been caused by an interaction between the drug and the polymeric blend, resulting in a reduction in purity and crystal lattice properties.

### 3.4. SEM Analysis

**Figure 6:**
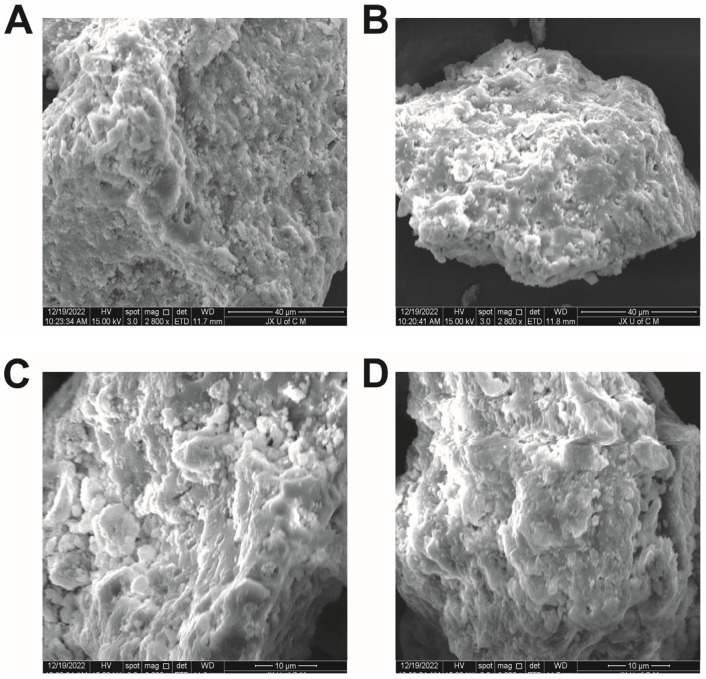
SEM micrographs of synthesized hydrogel at (**A**) 2800× from one angle, (**B**) 2800× from another angle, (**C**) 6000× from one side, and (**D**) 6000× from another side.

According to SEM micrographs, the hydrogel system exhibits rough, irregular, and porous structures, containing macropores and micropores (Figure 6). Hydrogel networks have porous surfaces, which permit the passage of water directly through the interstitial spaces, which allows fluid to diffuse through the network and enhances swelling capacity, as well as providing a suitable environment for the encapsulation and transportation of drugs. Water is taken up by the hydrogel network through the pores on the surface, resulting in the network absorbing a large volume of aqueous fluid. Similarly, a polymeric network’s stability is determined by solid mass and surface, as demonstrated by the hydrogel system [52].

### 3.5. Mechanical Properties and Drug Loading

Table 2 shows hydrogel mechanical properties, such as their tensile strength (TS) and elongation-at-break (EAB), which are important parameters to consider when designing a drug delivery system. The increase in EGDMA increased the tensile strength [53]. As AMPS content increases, the gel’s mechanical strength will decrease, possibly as a result of increasing electrostatic forces or osmotic pressure. Furthermore, synthetic polymer CP was incorporated into hydrogels for improved gelation and mechanical properties. The swelling of hydrogels affects its drug loading and release properties. So, hydrogels should have a sufficient mechanical strength so that, after higher swelling and drug loading, it is able to retain its structural integrity. If the crosslinking density is too high and hydrogels are mechanically too strong, then these factors will affect its swelling characteristics, and, therefore, lower drug loading will occur. Therefore, ideally, hydrogels used for drug delivery purposes should be evaluated for desired characteristics by varying the material ratios of the formulation and analyzing their effects on its swelling, drug loading, and release.

### 3.6. Sol–Gel Analysis

**Figure 7:**
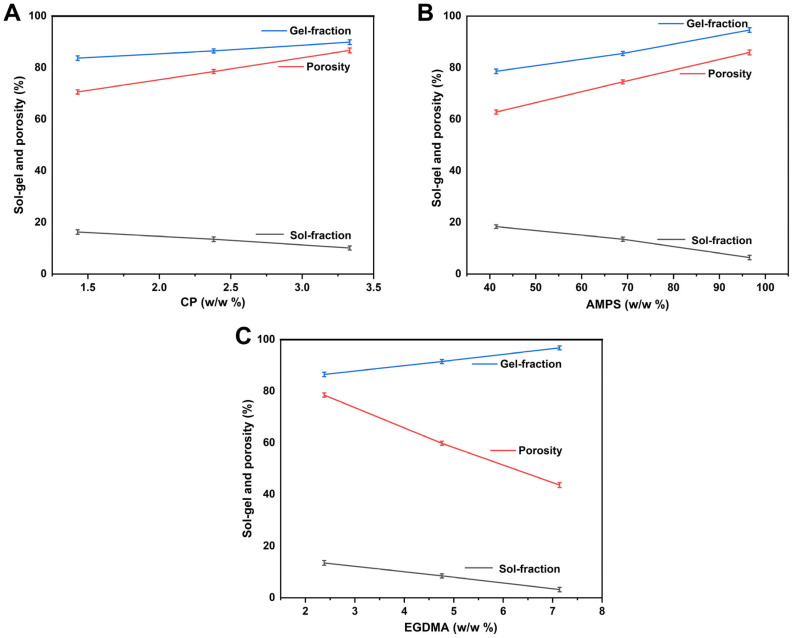
Influence of different concentrations of (**A**) CP, (**B**) AMPS, and (**C**) EGDMA on the sol–gel and porosity properties of CP-*g*-AMPS hydrogels.

Hydrogels are made when a polymerization reaction occurs, which results in a chain reaction, involving monomers, crosslinking agents, and polymers, and the part of the hydrogel that is not crosslinked is known as the “sol fraction”, while that which is crosslinked is called the “gel fraction”. The sol fraction occurs when high quantities of a component are used in hydrogels, and these parts remain uncrosslinked as a result of an insufficient number of reactive sites in the polymerization process. This requires the determination of the degree of crosslinking within the hydrogel as well as the degree of uncrosslinking within the hydrogel. Hydrogel formulations were all tested for sol–gel fractions (Figure 7A–C). As a result of the material ratio, the gel fraction percentage ranges from 83.70 to 96.80 percent. AMPS are hydrophilic monomers, and, as their concentration increases, more room is provided for chemical reactions, resulting in a higher gel fraction. A crosslinking agent, such as EGDMA, induces gel formation, and its content increases the gel fraction [54]. As the concentration of CP increases, the gel fraction increases as well, since free radicals are generated as a result of the rapid increase in polymer concentration. Thus, the gel fraction is also increased.

### 3.7. Porosity Evaluation

All of the hydrogels developed were tested for porosity. Porosity is a significant factor affecting hydrogel swelling properties, drug loading properties, and release characteristics. Therefore, it was essential to carefully monitor the porosity of the hydrogels in order to achieve the desired results. As pores become larger, swelling increases, which allows drugs to be loaded and released more effectively. Porosity is increased when a reaction mixture has a high viscosity, since this makes it more difficult for bubbles to escape. As a result, the porosity increases due to the establishment of interconnected channels. Porosity percentages ranged from 43.60% to 88.90% under different reagent ratios. The diameter of the pores decreases as the concentration of EGDMA increases due to the development of tight junctions and the increased cross-linking density, thereby reducing the network’s flexibility [55]. A higher concentration of AMPS improves porosity because the sulfonate groups are able to generate stronger electrostatic forces. AMPS contains an alkyl group that is hydrophobic, which can lead to a reduction in hydrogen bond interactions through the formation of hydrophobic microregions. Thus, the pore and network sizes of the hydrogel are enhanced during the preparation process, which is in accordance with other research results [56]. Porosity percentage increases with increasing CP concentration. This may be a result of the viscosity of the reaction mixture, causing bubbles to become trapped. Thus, interconnected channels are formed, leading to an increase in porosity.

### 3.8. Biodegradation Study

**Figure 8:**
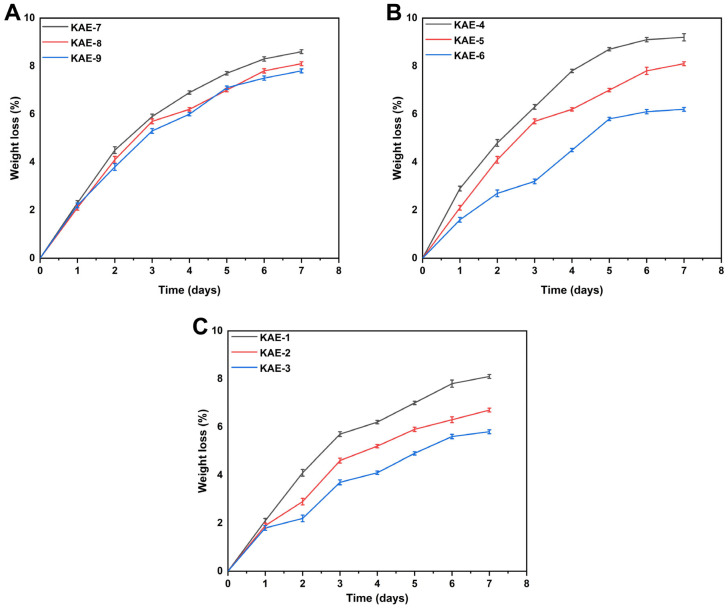
Influence of varying amounts of (**A**) CP (KAE-7,8,9), (**B**) AMPS (KAE-4,5,6), and (**C**) EGDMA (KAE-1,2,3) on the biodegradation of CP-*g*-AMPS hydrogels.

Figure 8 depicts the results of biodegradation tests performed to determine the hydrogel’s degradation rate. Various weight ratios will influence the degradation of gel in a significant manner. Hydrogels with different proportions demonstrated physical degradability of 5.8% to 9.2%. EGDMA decreased hydrogel degradation speed, possibly as a result of the establishment of new functional groups and tight junctions, which improved water gel content. Our study showed that, when CP concentration increased, the produced hydrogel degraded at a slower rate. It is possible that free radicals have been produced, resulting in hypergelation and, subsequently, the formation of a highly cross-linked hydrogel network. Consequently, cross-linking between hydrogel components increases hydrogel volume density, which results in a slow degradation of hydrogels [57].

### 3.9. Structural Parameters of Hydrogels

The structural parameters of synthesized hydrogels were calculated. Table 3 provides information regarding various structural parameters. Hydrogel parameters are important to calculate, as they indicate compatibility of the polymers with the solvents and, consequently, higher water uptake and holding capacity of the hydrogel. V2, S, and χ values improved as the EGDMA concentration was enhanced, indicating that gel structures were becoming tighter and stiffer, which reduced the mesh size of the hydrogel [58]. Thus, the mesh size plays an important role in the mechanical strength, degradation properties, and diffusion properties of the material, and it can be modified by modifying the percentage of crosslinker in the reaction mixture [59]. A similar decrease in Mc and N was observed when EGDMA concentrations were increased because crosslinking density increased. Additionally, Wang and colleagues found that, when methylene bisacrylamide (MBA) was used as a crosslinker to synthesize hydrogels and when its concentration was increased, the Mc value decreased, and the water absorption decreased, as well [60].

### 3.10. Swelling Behavior

**Figure 9:**
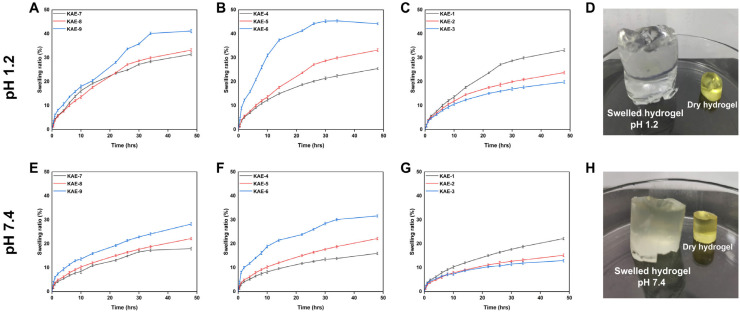
Effect of varying concentrations of components on hydrogels when swollen at pH 1.2 (**A**); CP, (**B**); AMPS, (**C**); EGDMA, (**D**); physical appearance and when hydrogels are swollen at pH 7.4 (**E**); CP, (**F**); AMPS, (**G**); EGDMA, and (**H**); physical appearance.

Hydrogels were synthesized using varying amounts of polymers/monomers and cross-linking agents to investigate the impact of their compositions upon swelling ratios across multiple pH media. Hydrogel composites swell when soaked in different media, since all polymers are hydrophilic and cross-linked. Figure 9 illustrates how swelling rates of hydrogels vary with pH over time. The synthesized hydrogels showed slightly higher swelling at a pH of 1.2 than at a pH of 7.4. The difference may be explained by the ionization of carboxylic acid groups to carboxylates at both pH. In the same manner, the −SO_3_H group also ionizes to the –SO_3_– group, regardless of the medium’s pH. Electrostatic repulsion between carboxylate and SO_3_– groups increases macromolecule expansion and reduces hydrogen bonding. Consequently, the formed hydrogel network swells more. Hydroxyl (–OH) functional groups of the hydrogels were ionized by water in the environment, resulting in increased swelling [61]. Swelling percentages for the hydrogel at a pH of 1.2 varied between 19.83% and 42.0%. Among these hydrogels, KAE-6 displayed the highest degree of swelling (42.20%), while KAE-3 displayed the lowest degree of swelling (19.83%) at a pH of 1.2. A swelling percentage of 12.95 to 31.6% was observed for the hydrogel at pH 7.4. KAE-6 had the highest swelling percentage (31.61%), while KAE-3 had the lowest swelling percentage (12.95%). Khalid et al. showed that CS-co-poly(AMPS) hydrogels show pH-independent swelling in different pH ranges, which is similar to our results [62]. Figure 9D, H shows the dried and swollen CP-g-AMPS hydrogels.

Figure 9A, E shows that water absorption (equilibrium swelling) increases with increasing CP content in KAE-7, KAE-8, and KAE-9 (1.428, 2.380, and 3.333 wt%, respectively), suggesting that there are more grafting sites available on the ends of the polymer chains. Low concentrations of CP assist monomer molecules in attaching to polymer reaction sites, and after monomer molecules have occupied the available reaction sites, they will be unable to attach further. This inhibition is due to the steric hindrance caused by the grafted chain and the redundant nature of the remaining monomer in the reaction system. In the case of excess monomers, they become homopolymers, which do not contribute to the absorption of water. As the polymer content increases, the homopolymer content decreases, providing a greater number of reaction sites for the monomer molecules and thus promoting water absorption. Additionally, increased CP content results in an increase in carboxylic acid groups, making the hydrogel more hydrophilic, thus increasing its swelling degree [39]. Since AMPS contains a large number of –CONH_2_ and –SO_3_OH groups, increasing the amount of monomer AMPS will strengthen the equilibrium swelling degree of hydrogel provided that other chemical components remain the same. The ionization of these chains increases their ability to interact with water molecules, so the higher the concentration, the greater the water uptake by hydrogels. When the concentration of EGDMA is increased, the swelling of the hydrogel is reduced. A higher concentration of EGDMA results in a higher packing density in hydrogels, which reduces their porosity. Water penetration into the hydrogel network is reduced as EGDMA concentration increases, which results in a reduction in swelling [63].

### 3.11. Release and Kinetic Modelling Analysis

**Figure 10:**
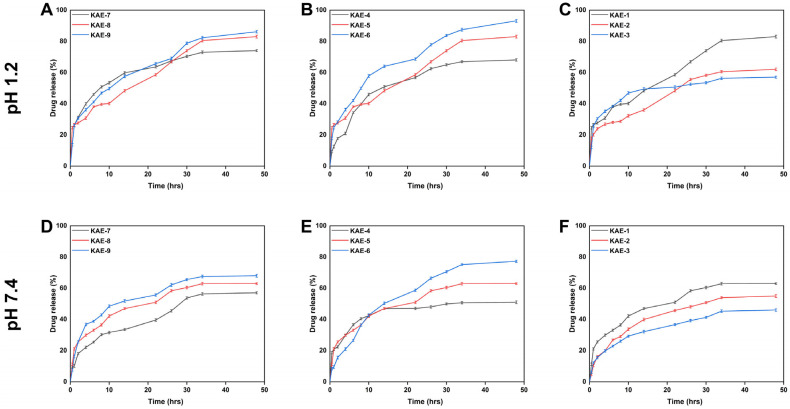
The drug release curve of CP-*g*-AMPS hydrogels and effect of components ((**A**); CP, (**B**); AMPS, and (**C**); EGDMA on release at pH 1.2 and at ((**D**); CP, (**E**); AMPS, and (**F**); EGDMA at pH 7.4.

In vitro drug release studies provide insight into how much active constituent escapes from the dosage form and becomes soluble over time [64]. Figure 10 shows the percentage of drug released in the pH 1.2 buffer varied between 57.03 and 92.13%. Drug release rates were highest for KAE-6 (92.13%) at pH 1.2, while they were the lowest for KAE-3 (57.03%). The release rate of drugs in a buffer with a pH of 7.4 ranged from 46.00% to 77.28%. KAE-6 (77.28%) achieved the highest drug release rate, while KAE-3 (46.0%) achieved the lowest after 48 h of testing. The rate at which EA is released varies with buffer pH, with the maximum release rate after 48 h in a buffer, with a pH of 1.2 being 92.13%. 

The osmotic pressure difference between the polymeric hydrogel disc and the surrounding water causes water molecules to diffuse into hydrogel discs when submerged in water. In this way, hydrogel is able to contain water within its structure. As a result of water diffusion, the hydrogel discs swell, allowing the loaded drug to be released through the channels within the discs [65]. The value of the regression coefficient that was near to one was utilized to determine which model was the most appropriate and provided the best explanation for the drug release data. According to Table 4, samples (KAE-7, KAE-8, and KAE-9) with different concentrations of CP followed Higuchi release kinetics because their regression coefficients were greater than zero and greater than first-order models. Similarly, samples (KAE-1, -2, and -3), containing differing amounts of the crosslinker EGDMA, followed Higuchi release kinetics, as indicated by high regression coefficient values. All samples, including those with varying amounts of AMPS (KAE-4, -5, and -6), exhibit diffusion-controlled drug release, as demonstrated by the regression coefficient (r) in the Higuchi models [66]. Normally, the diffusion is of two types in controlled drug delivery devices, namely, Fickian diffusion (0.45 < *n* < 0.5) and non-Fickian or anomalous diffusion, if the “*n*” value is between 0.5 and 1. Similarly, the developed hydrogels were used in cylindrical form, so the Fickian diffusion mechanism is described by 0.45 < *n* < 0.50. Overall, the hydrogels showed Fickian diffusion because the “*n*” values of all formulations ranged from 0.356 to 0.498 [67].

### 3.12. Antioxidation Analysis

**Figure 11:**
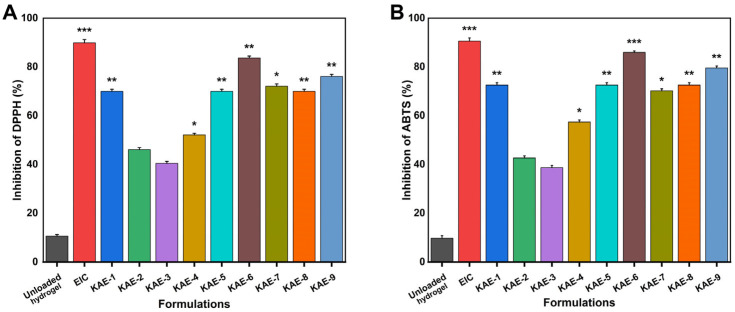
A comparison of the antioxidant properties of CP-*g*-AMPS hydrogels by using (**A**) DPPH and (**B**) ABTS (* p < 0.05, ** p < 0.01, *** p < 0.001).

Figure 11 shows the scavenging efficiency of DPPH and ABTS in the presence of hydrogels as an indicator of their antioxidant activity. As a result of their higher swelling and higher release, four formulations (KAE-6, KAE-9, KAE-1, and KAE-7) demonstrated greater antioxidant activity than the others. Antioxidant rates for DPPH ranged from 40.43% to 83.56%, while those for ABTS ranged from 38.69% to 85.96%. The antioxidant phenolic compound ellagic acid is found naturally in a number of fruits and vegetables, particularly pomegranates, raspberries, persimmons, black raspberries, peaches, strawberries, feathers, nuts (almonds, walnuts), and wine. Many studies have examined the potential health benefits of ellagic acid due to its antioxidant properties [68]. EIC-loaded hydrogels showed strong antioxidant properties under different proportions of material in our experiments.

### 3.13. Antibacterial Study

**Figure 12:**
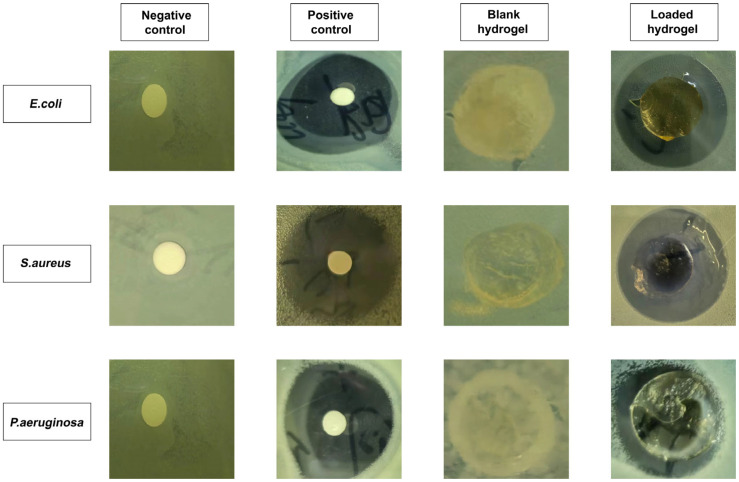
The zones of inhibition of samples against different bacterial strains.

Figure 12 illustrates the antibacterial activity of hydrogel against Gram-positive (*E. coli* and *S. aureus*) and Gram-negative (*P. aeruginosa*) bacteria. Negative control and blank hydrogel groups showed no zone formation, whereas clear zones were observed for the positive control (34 mm, 32 mm, and 35 mm) and the EIC-loaded hydrogel (14, 29, and 31 mm) against *P. aeruginosa*, *S. aureus*, and *E. coli*, respectively. There have been several studies that indicate that the broad-spectrum antibiotic cefepime is effective against both Gram-positive and Gram-negative bacteria [69].

Cefepime exhibited a smaller ZOI against Gram-negative bacteria compared to Gram-positive bacteria examined in the study. There could be a few different explanations for this behaviour, one of which being the composition of the cell wall of the bacteria. Gram-negative bacteria have a cell wall that is composed of three layers: the outer membrane, the peptidoglycan wall, and the cytoplasmic membrane. These layers are separated by an outer membrane [70]. The cell walls of Gram-positive bacteria are thick, but unlike Gram-negative bacteria, they do not have an outer membrane. Gram-negative bacteria are protected from the outside world by their outer membrane, which acts as a barrier against the effects of the external environment. Thus, the antibacterial effects of hydrogels and cefipime are much greater against Gram-positive bacteria than against Gram-negative bacteria.

The study has some limitations, such not being tested in vivo for the assessment of its toxicological and pharmacological effects, which are important for the evaluation of a new drug delivery system. A further investigation of the study will be conducted in the future.

## 4. Conclusions

In this study, HP-βCD and ellagic acid inclusion complexes (EIC) were developed, first, to increase the ellagic acid solubility. The results showed an entrapment efficiency of 83.96%, a drug loading of 27.31%, and a yield of 96.26% for ellagic acid in the EIC, which indicate a successful inclusion complexation process. The carbopol-934-g-AMPS controlled-release hydrogel was then synthesized through a free radical polymerization process. Different characterization techniques, such as FTIR, TGA, DSC, and XRD, provided confirmation of HP-βCD-ellagic acid inclusion complexes (EIC) formation, as well as successful incorporation of the drug into the hydrogel network. Additionally, morphological analysis by SEM revealed that the hydrogel has a rough and porous surface morphology. The hydrogels showed slightly higher swelling after 48 h at pH 1.2 (42.20%) compared with pH 7.4 (31.61%). Furthermore, the synthesized hydrogels released more drug at pH 1.2 (92.13%) than pH 7.4 (77.28%), suggesting that Fickian diffusion is the most likely mechanism for drug release. A substantial improvement in the mechanical properties and the release times of the drug was achieved by increasing the monomer concentration and polymer ratio. Additionally, the hydrogels exhibited high porosity (88.90%) and a high biodegradability (9.2% loss of weight in a week). A significant antioxidant effect was demonstrated by the developed hydrogels in the DPPH (inhibition rate 83.56%) and the ABTS testing (inhibition rate 85.96%). Moreover, hydrogels displayed excellent antibacterial properties against Gram-positive bacteria, such as *E. coli* (ZOI of 31 mm) and *S. aureus* (ZOI of 29 mm), as well as the Gram-negative bacteria *P. aeruginosa* (ZOI of 14 mm). In conclusion, the developed hydrogel system is capable of providing controlled delivery of both hydrophobic and hydrophilic drugs.

## Figures and Tables

**Table 1:** Feed composition (g/mL water) of CP-*g*-AMPS hydrogels.

Formulation Codes	Carbopol-934 (g)	APS/SHS (g)	AMPS (g)	EGDMA (g)
KAE-1	0.5/15	0.3/0.3/4	20/15	**0.5**
KAE-2	0.5/15	0.3/0.3/4	20/15	**1**
KAE-3	0.5/15	0.3/0.3/4	20/15	**1.5**
KAE-4	0.5/15	0.3/0.3/4	**12**/15	0.5
KAE-5	0.5/15	0.3/0.3/4	**20**/15	0.5
KAE-6	0.5/15	0.3/0.3/4	**28**/15	0.5
KAE-7	**0.3**/15	0.3/0.3/4	20/15	0.5
KAE-8	**0.5**/15	0.3/0.3/4	20/15	0.5
KAE-9	**0.7**/15	0.3/0.3/4	20/15	0.5

Note: Bold letters refer to increased feeding amounts.

**Table 2:** Hydrogels’ mechanical properties and EIC loading.

F. Codes	Thickness (mm)	TS (N/m^2^)	EAB (%)	EIC-Loaded per 1 g Hydrogel (g)
KAE-1	1.15 ± 0.005	0.502 ± 0.103	46.3 ± 1.056	0.487 ± 0.013
KAE-2	1.25 ± 0.007	0.676 ± 0.086	59.9 ± 1.687	0.417 ± 0.006
KAE-3	1.37 ± 0.004	1.107 ± 0.076	72.6 ± 1.551	0.329 ± 0.010
KAE-4	1.05 ± 0.003	0.816 ± 0.059	63.2 ± 2.922	0.460 ± 0.008
KAE-5	1.15 ± 0.005	0.502 ± 0.103	46.3 ± 1.056	0.487 ± 0.013
KAE-6	1.08 ± 0.008	0.413 ± 0.237	38.6 ± 1.687	0.596 ± 0.098
KAE-7	1.09 ± 0.004	0.517 ± 0.211	50.8 ± 2.398	0.499 ± 0.069
KAE-8	1.15 ± 0.005	0.502 ± 0.103	46.3 ± 1.056	0.487 ± 0.013
KAE-9	1.47 ± 0.007	0.581 ± 0.051	60.7 ± 1.201	0.518 ± 0.029

**Table 3:** Flory-Huggins network parameters of CP-*g*-AMPS hydrogels.

F. Codes	V_2,s_	χ	M_c_	M_r_	N	D × 10^−5^ (cm^2^ s^−1^)
KAE-1	0.030	0.510	3773.9	203.571	37.076	0.023
KAE-2	0.042	0.514	2356.7	224.307	21.013	0.031
KAE-3	0.050	0.517	1444.4	203.318	14.208	0.037
KAE-4	0.039	0.513	2358.4	201.461	23.412	0.028
KAE-5	0.030	0.510	3773.9	203.571	37.076	0.023
KAE-6	0.022	0.507	4363.8	204.517	42.674	0.015
KAE-7	0.031	0.510	2589.6	204.836	25.284	0.021
KAE-8	0.030	0.510	3773.9	203.571	37.076	0.023
KAE-9	0.024	0.508	3311.8	202.330	32.736	0.018

**Table 4:** Release kinetics of CP-*g*-AMPS hydrogels.

F. Codes	pH	Zero Order.		First Order		Higuchi Model		Korsmeyer-Peppas Model	
		K_o_ (h^−1^)	r^2^	K_1_ (h^−1^)	r^2^	K_2_ (h^−1^)	r^2^	r^2^	*n*
KAE-1	1.2	0.890	0.9640	0.011	0.9801	4.897	0.9942	0.9949 *	0.482
	7.4	0.576	0.9624	0.007	0.9714	3.218	0.9997	0.9998 *	0.495
KAE-2	1.2	0.644	0.9467	0.007	0.9599	3.609	0.9972	0.9976 *	0.484
	7.4	0.411	0.9424	0.005	0.9505	2.324	0.9970	0.9988 *	0.444
KAE-3	1.2	0.547	0.9372	0.006	0.9492	3.090	0.9956	0.9974 *	0.454
	7.4	0.372	0.9129	0.004	0.9215	2.138	0.9878	0.9983 *	0.360
KAE-4	1.2	0.687	0.9513	0.008	0.9650	3.839	0.9978 *	0.9977	0.498
	7.4	0.434	0.9421	0.005	0.9507	2.450	0.9969	0.9987 *	0.445
KAE-5	1.2	0.890	0.9640	0.011	0.9801	4.897	0.9942	0.9949 *	0.482
	7.4	0.576	0.9624	0.007	0.9714	3.218	0.9997	0.9998 *	0.495
KAE-6	1.2	1.406	0.8748	0.021	0.9294	8.087	0.9669 *	0.9963	0.401
	7.4	0.835	0.8536	0.010	0.8689	4.801	0.9918	0.9937 *	0.356
KAE-7	1.2	0.860	0.9462	0.011	0.9469	4.808	0.9954	0.9952 *	0.479
	7.4	0.445	0.9422	0.005	0.9510	2.515	0.9973	0.9993 *	0.435
KAE-8	1.2	0.890	0.9640	0.011	0.9801	4.897	0.9942	0.9949 *	0.482
	7.4	0.576	0.9624	0.007	0.9714	3.218	0.9997	0.9998 *	0.495
KAE-9	1.2	1.119	0.9638	0.015	0.9825	6.179	0.9919	0.9927 *	0.459
	7.4	0.746	0.9530	0.008	0.9652	4.206	0.9982	0.9992 *	0.437

* Represent higher r^2^ values.

## Data Availability

The data are contained within the article.
